# Role of glutathione, glutathione S-transferases and multidrug resistance-related proteins in cisplatin sensitivity of head and neck cancer cell lines.

**DOI:** 10.1038/bjc.1998.90

**Published:** 1998-02

**Authors:** M. J. Welters, A. M. Fichtinger-Schepman, R. A. Baan, M. J. Flens, R. J. Scheper, B. J. Braakhuis

**Affiliations:** Toxicology Division, TNO Nutrition and Food Research Institute, Zeist, The Netherlands.

## Abstract

Resistance to chemotherapy is a major problem in the treatment of patients with head and neck squamous cell carcinoma (HNSCC). Important factors involved are drug detoxification by glutathione (GSH) and reduced drug accumulation due to active transport out of the cell by so-called 'multidrug resistance-related proteins'. We have studied a panel of eight HNSCC cell lines showing differences in sensitivity to the anti-cancer drug cisplatin. Our previous studies indicated that the IC50 values were inversely correlated with the intracellular accumulation of platinum (Pt). In the present study, cellular GSH levels were found not to be related to the IC50 values. The expression levels of the enzymes glutathione S-transferase (GST) alpha, mu, and pi, the multidrug resistance-related proteins P-glycoprotein (P-gp), multidrug resistance-associated protein (MRP) and the lung resistance protein (LRP) were determined semiquantitatively by means of immunocytochemistry. The levels of the GSTs, P-gp and LRP were not found to be correlated with the IC50 values of the HNSCC cell lines. Surprisingly, however, an inverse correlation was found between MRP levels and IC50 values. The MRP expression levels were in agreement with the results of the MRP functional assay, based on the transport of calcein across the cell membrane as performed for two of the cell lines. Further studies should prove whether other pump mechanisms or DNA repair are involved in the cisplatin accumulation and the subsequent HNSCC cell growth inhibition.


British Journal of Cancer (1998) 77(4), 556–561
© 1998 Cancer Research Campaign

### Role of glutathione, glutathione S-transferases and multidrug resistance-related proteins in cisplatin sensitivity of head and neck cancer cell lines

**MJP Welters[1,2], AMJ Fichtinger-Schepman[1], RA Baan[1], MJ Flens[3], RJ Scheper[3] and BJM Braakhuis[2]**

[1]Toxicology Division, TNO Nutrition and Food Research Institute, PO Box 360, 3700 AJ Zeist, The Netherlands; Departments of [2]Otolaryngology and [3]Pathology, University Hospital, Vrije Universiteit, PO Box 7057, 1007 MB Amsterdam, The Netherlands

**Summary** Resistance to chemotherapy is a major problem in the treatment of patients with head and neck squamous cell carcinoma (HNSCC). Important factors involved are drug detoxification by glutathione (GSH) and reduced drug accumulation due to active transport out of the cell by so-called 'multidrug resistance-related proteins'. We have studied a panel of eight HNSCC cell lines showing differences in sensitivity to the anti-cancer drug cisplatin. Our previous studies indicated that the $IC_{50}$ values were inversely correlated with the intracellular accumulation of platinum (Pt). In the present study, cellular GSH levels were found not to be related to the $IC_{50}$ values. The expression levels of the enzymes glutathione S-transferase (GST) $\alpha$, $\mu$ and $\pi$, the multidrug resistance-related proteins P-glycoprotein (P-gp), multidrug resistance-associated protein (MRP) and the lung resistance protein (LRP) were determined semiquantitatively by means of immunocytochemistry. The levels of the GSTs, P-gp and LRP were not found to be correlated with the $IC_{50}$ values of the HNSCC cell lines. Surprisingly, however, an inverse correlation was found between MRP levels and $IC_{50}$ values. The MRP expression levels were in agreement with the results of the MRP functional assay, based on the transport of calcein across the cell membrane as performed for two of the cell lines. Further studies should prove whether other pump mechanisms or DNA repair are involved in the cisplatin accumulation and the subsequent HNSCC cell growth inhibition.

**Keywords:** glutathione; glutathione S-transferase; multidrug resistance; cisplatin; head and neck cancer

Cisplatin shows activity in patients with advanced head and neck squamous cell carcinoma (HNSCC). The response of these tumours differs between patients, but a good initial response is generally seen upon cisplatin chemotherapy in 20–50% of the cases (Vokes et al, 1993). Nevertheless, treatment does not lead to an increased survival as a consequence of a lack of response or its short duration.

Various mechanisms have been proposed to explain resistance to cisplatin (Hayes and Wolf, 1990). The role of glutathione (GSH) in cisplatin resistance seems to be important as cells with in vitro acquired resistance often show elevated levels of GSH compared with the parental cells (Meijer et al, 1992; Goto et al, 1995). Glutathione S-transferases (GST) are enzymes that catalyse the conjugation of cisplatin to GSH. The cisplatin–GSH complex has been proposed to be ejected from the cell in an ATP-dependent fashion by the glutathione S-conjugate (GS-X) export pump (Ishikawa and Ali-Osman, 1993; Goto et al, 1995).

ATP-dependent transport systems, referred to as pumps, are proposed to be responsible for resistance to multiple drugs, i.e. multidrug resistance (MDR) (Biedler, 1992). Two important MDR-associated membrane-bound proteins are P-glycoprotein (P-gp), encoded by the *MDR1* gene, and the multidrug resistance-associated protein (MRP) (Broxterman et al, 1995; Nooter et al, 1995). P-gp and MRP actively transport a wide range of substrates across membranes into vesicles and out from the cell. A number of substrates are transported by MRP after conjugation to GSH (Müller et al, 1994). Another MDR-associated protein, recently discovered, is the lung resistance protein (LRP), possibly mediating intracellular transport (Scheper et al, 1993; Scheffer et al, 1995). Although cisplatin is not known to induce MDR itself, MDR-induced cells can become cross-resistant to cisplatin (Loe et al, 1996). The possible involvement of MDR in the response to platinum (Pt)-based treatments has been reported in a panel of 61 human cell lines of eight different cancer types (Izquierdo et al, 1996*a*) and in patients with ovarian cancer (Izquierdo et al, 1995). HNSCC was not included in these studies, although other studies have shown that HNSCC cells can express P-gp (Kelley et al, 1993), MRP (Nooter et al, 1995), as well as LRP (Izquierdo et al, 1996*b*).

Using a panel of eight HNSCC cell lines that differ in cisplatin sensitivity, we were able to show an inverse correlation between $IC_{50}$ values and Pt accumulation (Welters et al, 1997). To investigate the underlying mechanism of differences in cisplatin sensitivity and Pt accumulation, we presently report on the GSH levels and the expression levels of the GST isoenzymes as well as of the MDR proteins P-gp, MRP and LRP. In addition, the MRP activity was determined.

*Received 22 May 1997*
*Revised 20 August 1997*
*Accepted 21 August 1997*

*Correspondence to:* BJM Braakhuis

### MATERIAL AND METHODS

### Tumour cell lines

Human HNSCC cell lines UM-SCC-11B, UM-SCC-14C, UM-SCC-22A, UM-SCC-22B and UM-SCC-35 were described by Carey et al (1990). These cell lines were established from fresh tumour biopsies. The same holds for cell lines 92VU040T and 93VU120T (Hermsen et al, 1996). VU-SCC-OE was established in our laboratory from a HNSCC xenograft (Welters et al, 1997). Cells were routinely cultured in Dulbecco's modified Eagle medium (Life Technologies, Gibco BRL, Breda, The Netherlands) supplemented with 5% heat-inactivated fetal calf serum (Flow, Irvine, UK), 50 U $ml^{-1}$ penicillin and 50 μg $ml^{-1}$ streptomycin (Life Technologies).

### Cisplatin treatment

HNSCC cells at subconfluency were treated with cisplatin for 72 h, washed twice with phosphate-buffered saline (PBS) and harvested by use of trypsin. The $IC_{50}$ data of the HNSCC cells, i.e. the concentration of the drug causing 50% of growth inhibition compared with that of untreated control cells, have been published before (Welters et al, 1997) and were determined with the sulphorhodamine B (SRB) assay (Braakhuis et al, 1993).

### GSH content in cultured human HNSCC cells

GSH was measured in untreated and cisplatin-treated HNSCC cells by high-performance liquid chromatography (HPLC) combined with precolumn derivatization with orthophthaldehyde and fluorometric detection (Neuschwander-Tetri and Roll, 1989). GSH levels per cell line were measured in two or three independent samples of cells cultured at subconfluency.

### Immunocytochemical staining of GST isoenzymes

Expression of the GST isoenzymes α, μ and π was analysed using the immunoperoxidase staining method described by Bongers et al (1995). HNSCC cells were deposited on glass slides with a cytospin centrifuge, fixed with methanol for 10 min and washed with PBS. A 30-min preincubation was performed with 2% normal swine serum (Dako, Copenhagen, Denmark) diluted in PBS containing 1% bovine serum albumin (BSA; Sigma, St Louis, MO, USA), followed by incubation with rabbit antisera directed against GST-α, -μ and -π respectively (antisera diluted 1:1 in PBS/1% BSA; NovoCastra, Newcastle upon Tyne, UK). The preparations were washed three times with PBS (5 min each), and treated for 10 min with 0.006% hydrogen peroxide in methanol to inhibit endogenous peroxidase activity, followed by three washings with PBS. The slides were then incubated for 30 min with swine anti-rabbit biotin conjugate (diluted 1:500 in PBS/1% BSA; Dako) and washed again three times with PBS. After a further incubation for 60 min with avidin–biotin complex (Vectastain ABC-kit, Vector Laboratories, Burlingame, CA, USA), and three wash steps, antibody binding was visualized by incubation with 4 mg (v/v) of 3,3′-diaminobenzidine tetrahydrochloride (Sigma) and 0.02% (v/v) hydrogen peroxide in PBS for 3–5 min. The slides were rinsed with tap water, counterstained with haematoxylin (Merck, Darmstadt, Germany), and finally mounted with Kaiser's glycerin gelatin (Merck). As a negative control, slides were incubated as described above except that the primary antibody was replaced by PBS/1% BSA or mouse IgG antibody. In two independent experiments, all cell lines were stained simultaneously with the various antibodies.

**Table 1** Parameters determining cisplatin sensitivity in cultured HNSCC cells

| Cell line | $IC_{50}$ value[a] | Pt accumulation[b] | GSH levels[c] |
|---|---|---|---|
| UM-SCC-35 | 0.9 ± 0.8 | 159 ± 93 | 10.2 ± 1.8 |
| UM-SCC-22B | 1.2 ± 0.3 | 149 ± 34 | 5.0 ± 2.3 |
| UM-SCC-22A | 1.3 ± 0.3 | 109 ± 9 | 6.3 ± 2.6 |
| 92VU040T | 2.0 ± 0.5 | 67 ± 7 | 11.2 ± 3.1 |
| UM-SCC-11B | 2.2 ± 0.6 | 126 ± 9 | 7.5 ± 1.1 |
| VU-SCC-OE | 2.3 ± 0.9 | 566 ± 317 | 7.1 ± 0.7 |
| UM-SCC-14C | 2.7 ± 0.7 | 81 ± 10 | 2.0 ± 1.2 |
| 93VU120T | 2.8 ± 1.0 | 89 ± 10 | 6.1 ± 0.7 |

[a]The sensitivity to cisplatin was determined by a cell proliferation (SRB) assay. The $IC_{50}$ value, the concentration of the drug causing 50% growth inhibition after a 72-h treatment, is given in μM cisplatin. These results were obtained in a previous study and were reported to be significantly correlated with Pt accumulation data when those of cell line OE were omitted (Welters et al, 1997). [b]The total amount of Pt accumulated in the cells (expressed as pmol Pt per $10^6$ cells), after treatment with 10 μM of cisplatin for 72 h, was determined with AAS in a previous study (Welters et al, 1997). [c]Glutathione (GSH) levels (fmol per cell) were determined by HPLC according to Neuschwander-Tetri et al (1989)

### Immunocytochemical staining of P-gp, MRP and LRP

The HNSCC cells were cultured until subconfluency and harvested onto cytocentrifuge slides, which were stored at −20°C until analysis. Immunocytochemistry was performed as described by Izquierdo et al (1996*b*). In short, after thawing the cytocentrifuge preparations were acetone-fixed (10 min) before preincubation with 2% normal rabbit serum for 15 min (Dako). Then, slides were incubated for 60 min at room temperature with one of the following monoclonal antibodies (MAb): mouse MAb JSB-1 (1:100 of 10 μg $ml^{-1}$) against P-gp, mouse MAb MRP-m6 (1:25 of 1 μg $ml^{-1}$) and rat MAb MRP-r1 (1:1500 of 1 μg $ml^{-1}$) against MRP, rat MAb LRP-56 (1:500 of 0.5 μg $ml^{-1}$) and LMR-5 (1:500 of 0.5 μg $ml^{-1}$) both directed against LRP. These antibodies are available from Sanbio, Uden, The Netherlands. After washing with PBS for 15 min, the slides were incubated for 60 min with rabbit anti-mouse biotin (1:150; Zymed Laboratories, San Francisco, CA, USA) or rabbit anti-rat biotin (1:100) conjugate (Dako), washed and incubated with streptavidin coupled to horseradish peroxidase (1:500; Zymed Laboratories) for 60 min. All dilutions were in PBS with 1% BSA. The washed cells were finally stained with amino-ethyl-carbazole (ICN Biochemicals, Aurora, OH, USA) for 5 min and counterstained with haematoxylin (Merck). As a negative control, irrelevant IgG or PBS was used instead of the primary antibody. Positive controls for the expression of each of the proteins were KB-8-5 cells for P-gp (Izquierdo et al, 1995), GLC4/ADR cells for MRP and SW-1573/2R120 cells for LRP (Scheper et al, 1993; Flens et al, 1994; Broxterman et al, 1996). Immunohistochemical staining of the cell lines was performed in two independent experiments (I and II) and all slides of each experiment were stained simultaneously.

### Evaluation of immunocytochemical staining

The evaluation was performed using light microscopy on coded slides. Scoring of each immunocytochemical experiment was performed blindly and independently by three observers. The number of cells that stained very strong (+ + +), strong (+ +), intermediate (+) or not (–) was expressed as a percentage of the total number of cells investigated. The semiquantitative staining index

© Cancer Research Campaign 1998

**Table 2** Correlation between $IC_{50}$ values of the HNSCC cell lines and the expression levels of GST and MDR-related proteins

| Marker | Antibody designation | Experiment I | | Experiment II | |
|---|---|---|---|---|---|
| | | *r*-value | *P*-value | *r*-value | *P*-value |
| GST-α | GSTalpha | –0.02 | 0.95 | –0.05 | 0.89 |
| GST-μ | GSTmuM2 | 0.71 | 0.06 | 0.41 | 0.31 |
| GST-π | GSTpi | 0.57 | 0.13 | 0.57 | 0.12 |
| P-gp | JSB-1 | –0.50 | 0.19 | –0.52 | 0.17 |
| MRP | MRP-m6 | –0.79 | 0.04 | –0.71 | 0.05 |
| | MRP-r1 | –0.83 | 0.03 | –0.83 | 0.03 |
| LRP | LRP-56 | –0.45 | 0.23 | –0.47 | 0.22 |
| | LMR-5 | –0.60 | 0.12 | –0.45 | 0.29 |

The relations between the $IC_{50}$ values and the expression levels of the various markers, which were recognized and visualized by antibodies, were determined in two independent experiments. The Spearman's rank correlation coefficients (*r*-values) and significancies (*P*-values) are given.

of each group was calculated as the product of this percentage and the staining intensity. The latter was estimated on a scale of 1 (+) to 3 (+ + +). The variation in scores between the three observers, expressed as a coefficient of variation, i.e. the s.d. as a percentage of the mean, was always less than 30%. Intraobserver variation of scoring was tested and was proved to be less than 20%.

### Functional MRP test

The HNSCC cell lines UM-SCC-14C that showed a low sensitivity to cisplatin and UM-SCC-35, the most sensitive cell line of our panel, were analysed in two independent experiments for the presence of functional MRP as described by Feller et al (1995). Briefly, about $0.5 \times 10^6$ cells were allowed to take up calcein-acetoxymethylester (calcein-AM) by incubation in 0.5 μM of this dye for 10 min at 37°C. They were washed and subsequently incubated in fresh medium with or without the MRP inhibitor probenecid (1.0 mM, Sigma) for 0, 10 or 60 min. The efflux was stopped by centrifugation of the cells and addition of ice-cold culture medium. In this assay, the non-fluorescent dye calcein-AM is converted by intracellular esterases to the fluorescent calcein. The calcein can be exported by active MRP, which can be prevented by the use of the MRP inhibitor. The intracellular calcein is then analysed using FACScan flow cytometry (Becton Dickinson Medical Systems, Sharon, MA, USA). The human small-cell lung cancer cell line GLC4, which is MRP negative, and its MRP-overexpressing subline GLC4/ADR were used as controls (Feller et al, 1995).

### Statistical analysis

Correlations between the various cellular parameters and the $IC_{50}$ values of the cultured HNSCC cells were determined by Spearman's rank correlation test; the correlation coefficients (*r*-values) and the *P*-values (two-sided) were calculated. Only correlations with *P*-values of 0.05 or below were considered to be significant.

### RESULTS

The efficacy of cisplatin treatment in a panel of eight human HNSCC cell lines was compared with GSH, GST and MDR-related

**Figure 1** The relationship between the sensitivity to cisplatin, expressed as $IC_{50}$ value, and the semiquantitative staining index of MRP using either antibody MRP-m6 (●; solid line) or MRP-r1 (△; dashed line) as determined in the two independent experiments I (A) and II (B)

protein levels. As previously published, the $IC_{50}$ values varied about three-fold between the cell lines and showed a significant inverse correlation with the Pt accumulation in these cells when data of cell line OE (derived from a previously irradiated patient) were omitted (Table 1; Welters et al, 1997).

### GSH levels in untreated and cisplatin-treated cells

The total levels of GSH in the eight HNSCC cell lines varied between 2.0 fmol per cell for UM-SCC-14C and 11.2 fmol per cell for 92VU040T (Table 1). No correlation was found with the $IC_{50}$ values or the cellular Pt content. The GSH level in the VU-SCC-OE cells appeared to be within the range of the other cell lines and could, therefore, not explain the moderate sensitivity of this cell line and its high Pt content. To study possible induction of GSH by cisplatin treatment, cell lines UM-SCC-14C, VU-SCC-OE and UM-SCC-35, showing differences in $IC_{50}$ values, were treated with 0.1 and 1.0 μM cisplatin during 5 and 24 h. In these treated cells, a small increase of GSH levels was found compared with the untreated cells (data not shown). However, this induction of GSH was slightly different among the cell lines. Therefore, these differences in cisplatin-induced GSH levels cannot be held responsible for the variation in $IC_{50}$ values found for these cell lines.

### Expression of the GST isoenzymes

In all HNSCC cell lines, the presence of the three isoforms GST-α, GST-μ and GST-π could be demonstrated by immunocytochemical

© Cancer Research Campaign 1998

**Table 3** Activity of MRP protein

| Cell line | Duration of calcein efflux | |
|---|---|---|
| | $t$ = 10 min[a] | $t$ = 60 min[b] |
| UM-SCC-35 | 1.54 ± 0.05 | 1.47 ± 0.21 |
| UM-SCC-14C | 1.02 ± 0.03 | 1.22 ± 0.15 |
| GLC4 | 0.94 | 1.10 ± 0.03 |
| GLC4/ADR | 2.0 | 3.05 ± 0.39 |

The effect of the MRP inhibitor (1.0 mM probenecid) is expressed as the ratio of calcein accumulation in the presence of this modulator divided by that in the absence of probenecid, measured after a duration of [a]10 min or [b]60 min of calcein efflux. GLC4 was included as negative and GLC4/ADR as positive control cells. Experiments were performed in duplicate, except for the 10 min incubation experiment of GLC4 and GLC4/ADR.

staining. Over 90% of the cells of each line were positive for GST-π. The staining percentages for the other two GST isoenzymes were lower and varied considerably between the cell lines. For GST-α the percentage of positively stained cells varied between 15% and 100%, whereas for GST-μ it varied from 3% to 100%. The calculated staining indices (see Material and methods) differed between the cell lines and between the three isoforms of GST, but none of these correlated with the $IC_{50}$ values (Table 2), neither with the Pt accumulation in these cells after 72 h of exposure to cisplatin nor with the GSH levels determined in the untreated cells.

### Expression of MDR-related proteins

The expression level of the MDR protein P-gp, visualized by use of antibody JSB-1, was expressed in all HNSCC cell lines tested, with staining index ranging from 128 (93VU120T) to 262 (UM-SCC-35). The levels of MRP, measured with specific mouse and rat antibodies, were also different for the various HNSCC lines. The UM-SCC-14C cells appeared to be stained very weakly or not at all, indicating that MRP levels were relatively low. The data obtained with the mouse and rat anti-MRP antibodies were correlated significantly, resulting in a correlation coefficient ($r$-value) of 0.72 ($P$ = 0.05) in the first and $r$ = 0.80 ($P$ = 0.04) in the second experiment. The staining index of LRP-56, a measure of the presence of LRP, ranged from 107 for UM-SCC-14C cells to 202 for cell line UM-SCC-35. With the LMR-5 antibody, which also recognizes LRP, similar variations in staining level were observed. The results obtained with these two antibodies recognizing LRP did significantly correlate in the two experiments ($r$ = 0.63, $P$ = 0.02 and $r$ = 0.72, $P$ = 0.05).

To find out whether the levels of these three membrane proteins have an effect on the sensitivity of the cells to cisplatin, the relationships between these levels and the $IC_{50}$ values were determined. A significant inverse correlation was found in the first experiment between the $IC_{50}$ values and MRP, as indicated by the staining index of MRP-m6 ($r$ = –0.79, $P$ = 0.04) and of MRP-r1 ($r$ = –0.83, $P$ = 0.03) (see Fig. 1A and Table 2). The second experiment (Fig 1B) confirmed this finding, showing a significant correlation of $IC_{50}$ values with the MRP-m6 staining index ($r$ = –0.71, $P$ = 0.05) as well as with the MRP-r1 staining index ($r$ = –0.83, $P$ = 0.03). No correlation was found between the $IC_{50}$ values and the P-gp or the LRP levels (Table 2).

Whether or not the total amount of Pt accumulated in the HNSCC cells (with the exception of VU-SCC-OE) is correlated with the expression levels of MRP as visualized with antibody MRP-m6 is not quite clear. In the first experiment, the correlation was found not to be significant ($r$ = 0.68, $P$ = 0.09), but in the second experiment it was significant ($r$ = 0.89, $P$ = 0.03). The same holds true for the results obtained with the other MRP-recognizing antibody, showing a significant correlation between Pt accumulation levels and MRP-r1 staining results, in experiment I ($r$ = 0.77, $P$ = 0.05) and no significance in experiment II ($r$ = 0.61, $P$ = 0.13).

### Functional MRP test

Cell lines UM-SCC-14C and UM-SCC-35, which differed significantly in MRP expression, were used to determine if the established differences in the levels of MRP were indicative for differences in the MRP activity in the cells. The results of the assay are given in Table 3. The UM-SCC-14C cells showed hardly any activity of the MRP pump, whereas the cells of line UM-SCC-35 appeared to have functional MRP after 10-min and 60-min treatments determined with the MRP inhibitor probenecid. This is in agreement with the immunocytochemical staining results, in which the presence of MRP could not be demonstrated in UM-SCC-14C cells whereas a relatively high expression was observed in UM-SCC-35.

### DISCUSSION

Our data indicate a minor role for GSH as a determining factor of the differences in sensitivity to cisplatin of the presently studied HNSCC cell lines. An inverse correlation between the GSH levels and cisplatin sensitivity has been reported for cell lines of various tumour types, thereby partly explaining the resistance found (Mistry et al, 1991; Meijer et al, 1992). It should be noted that we studied cell lines that were not treated in vitro to obtain acquired resistance. Because Yellin et al (1994) reported that the GSH levels in HNSCC cells can be up-regulated during cisplatin treatment, the GSH levels were also determined in the cell lines UM-SCC-14C, UM-SCC-35 and VU-SCC-OE after incubation with cisplatin during various time periods. As a result, only small increases in GSH levels occurred, but this did not lead to correlations with the $IC_{50}$ values.

In the detoxification system GSH/GST, GSTs catalyse the binding of electrophilic components to GSH. Three isoforms of GST can be distinguished in humans namely π (acidic), μ (neutral) and α (basic). Expression of GSTs and MDR-related proteins was studied by immunocytochemistry. This was known to be a reliable method because for these proteins a correlation was found with the outcome of Western blots, immunoprecipitation analyses and the determination of the corresponding mRNA levels (Flens et al, 1994; Nooter et al, 1995; Scheffer et al, 1995). Comparison of the GST staining indices of our eight HNSCC lines with the $IC_{50}$ values of these lines revealed no correlation (see Table 2), which is in agreement with the results of Yellin and colleagues (1994) for a panel of 14 HNSCC lines. It cannot be excluded that the other factors in the GSH-associated detoxification system play a role in cisplatin sensitivity; this includes the enzymes glutathione peroxidase, glutathione synthetase, glutathione reductase and dipeptide gamma-glutamyl-cysteine (Kramer et al, 1988; Kurokawa et al, 1995).

The importance of the MDR proteins in the efficacy of Pt-containing chemotherapy has recently been reported for leukaemia cells and colon carcinomas (Ishikawa et al, 1994; 1996). In the present study, no significant correlation was found between P-gp

© Cancer Research Campaign 1998

expression levels and the $IC_{50}$ values, suggesting no direct involvement of P-gp in the in vitro response of HNSCC cells to cisplatin. A significant, but inverse correlation was found between MRP and the $IC_{50}$ values (Table 2). MRP was detected on the membranes of HNSCC cells, as well as inside these cells, with the two antibodies MRP-m6 and MRP-r1. The staining results obtained with MRP-m6 were significantly correlated with those of MRP-r1, which is in line with results reported by Izquierdo et al (1996*a*). It should be noted that these antibodies do not cross-react with human MDR1 and MDR3 P-gps (Flens et al, 1994). A high expression of MRP is usually determined in cell lines with acquired resistance (Müller et al, 1994; Brock et al, 1995). It is thought that MRP is a GS-X pump (Müller et al, 1994; Loe et al, 1996; O'Brien and Tew, 1996), which is present on vesicles and/or the plasma membrane (Nooter et al, 1995). The unexpected finding in our panel of HNSCC that the correlation of MRP with the $IC_{50}$ data was inverse (Fig 1), and thus positive with sensitivity, and that high cellular Pt levels were associated with high MRP expression levels cannot be attributed to less active MRP because we provided evidence that the MRP was indeed active as determined by the functional MRP assay (see Table 3). These data implicate that in HNSCC cells the GS-X pump activity, i.e. transporting GSH-conjugated cisplatin out of cells, may not be the major function of MRP. This is in agreement with the results of De Vries et al, (1995). A possible explanation for the unexpected relation of higher sensitivity in the presence of more MRP may be that endogenous metabolites conjugated to GSH are extruded from the cell, whereas cisplatin is counter-transported. Another hypothesis is the regulation of endogenous (ion) channels, and possibly other transporters, by MRP as described by Loe and colleagues (1996), which can lead to an increase of the influx of cisplatin into the cells and eventually into the nucleus. As a consequence, the levels of DNA-bound Pt will increase. The involvement of other as yet undefined transport mechanisms in the sensitivity to cisplatin of the HNSCC lines under study also cannot be ruled out. Possible candidates for alternative pumps are the human canalicular multispecific organic anion transporter (cMOAT), also designed as MRP2, which has been described to be overexpressed in the cisplatin-resistant human head and neck cancer KB cell line (Taniguchi et al, 1996) and the SQM1 protein, which is present at reduced levels in HNSCC resistant to methotrexate and cisplatin (Bernal et al, 1990).

High expression of the non-P-gp LRP protein in acute myeloid leukaemia and ovarian carcinoma has been associated with a poor response to chemotherapy, such as cisplatin treatment (Izquierdo et al, 1995; Scheffer et al, 1995). In our HNSCC cell lines LRP was detectable, which is in agreement with the earlier published finding that this protein is present in epithelial cells (Scheffer et al, 1995) and head and neck tumours (Izquierdo et al, 1996*b*). No correlation was found between LRP expression levels and the $IC_{50}$ values (Table 2), or with the Pt accumulation data. These results are in contrast with those found by Izquierdo and colleagues (1996*a*), who showed a predictive value of LRP for in vitro sensitivity to several types of drugs, among which also cisplatin, in a number of cancer types. Their study, however, did not include HNSCC. Our results in eight HNSCC cell lines indicate that pump mechanisms other than LRP control the response of this cancer type to cisplatin. The importance of DNA damage recognition proteins in DNA repair and the nucleotide excision repair system in repairing cisplatin-DNA damage has been reviewed by Hill (1996). It is clear also that other unknown factors may contribute to the differences in sensitivity to cisplatin in our HNSCC cell lines. In addition, it is obvious that intrinsic sensitivity to drugs is a very complex phenomenon that needs further investigation.

In conclusion, an inverse correlation was found between the $IC_{50}$ values of HNSCC cell lines, obtained after 72 h of cisplatin treatment, and their expression level of the MDR-associated membrane-bound protein MRP. In addition, the indications for a positive relation between Pt accumulation and MRP expression levels suggest that MRP plays a role in transport of cisplatin into or inside the HNSCC cells.

### ABBREVIATIONS

BSA, bovine serum albumin; calcein-AM, calcein acetoxymethylester; GSH, glutathione; GST, glutathione S-transferase; GS-X, glutathione S-conjugate export pump; HNSCC, head and neck squamous cell carcinoma; $IC_{50}$ value, concentration of drug that inhibits cell growth to 50% of control growth; LRP, lung resistance protein; MAb, monoclonal antibody; MDR, multidrug resistance; MRP, multidrug resistance-associated protein; P-gp, P-glycoprotein; Pt, platinum.

### ACKNOWLEDGEMENTS

We thank H. Joenje (Department of Human Genetics, Vrije Universiteit, Amsterdam) for providing cell lines 92VU040T and 93VU120T; Professor Dr WJF van der Vijgh and Dr HJ Broxterman (Department of Oncology, Vrije Universiteit, Amsterdam) for critical reading of the manuscript; Dr T Teerlink (Central Chemical Laboratory, Vrije Universiteit, Amsterdam) for his advice. AJ Jacobs-Bergmans, JE Pankras, DCR Währer, S de Jong and AB Schroeijers are acknowledged for their technical assistance. This study was financially supported by the Dutch Cancer Society (Grant MBL 92-74).

### REFERENCES

Bernal SD, Speak JA, Boeheim K, Dreyfuss AI, Wright JE, Teicher BA, Rosowsky A, Tsao S-W and Wong Y-C (1990). Reduced membrane protein associated with resistance of human squamous carcinoma cells to methotrexate and cisplatinum. *Mol Cell Biochem* **95**: 61–70

Biedler JL (1992) Genetic aspects of multidrug resistance (review). *Cancer* **70**: 1799–1809

Bongers V, Snow GB, de Vries N, Cattan AR, Hall AG, Van Der Waal I and Braakhuis BJM (1995) Second primary head and neck squamous cell carcinoma predicted by the glutathione S-transferase expression in healthy tissue in the direct vicinity of the first tumor. *Lab Invest* **73**: 503–510

Braakhuis BJM, Jansen G, Noordhuis P, Kegel A and Peters GJ (1993) Importance of pharmacodynamics in the *in vitro* antiproliferative activity of the antifolates methotrexate and 10-ethyl-10-deazaaminopterin against human head and neck squamous cell carcinoma. *Biochem Pharmacol* **46**: 2155–2161

Brock I, Hipfner DR, Nielsen BS, Jensen PB, Deeley RG, Cole SPC and Sehested M (1995) Sequential coexpression of the multidrug resistance genes MRP and mdr1 and their products in VP-16 (etoposide)-selected H69 small lung cancer cells. *Cancer Res* **55**: 459–462

Broxterman HJ, Giaccone G and Lankelma J (1995) Multidrug resistance proteins and other drug transport-related resistance to natural product agents. *Curr Opin Oncol* **7**: 532–540

Broxterman HJ, Lankelma J and Pinedo HM (1996) How to probe clinical tumour samples for p-glycoprotein and multidrug resistance-associated protein. *Eur J Cancer* **32A**: 1024–1033

Carey TE, Wolf GT, Baker SR and Krause CJ (1990) Cell surface antigen expression and prognosis. In *Head and Neck Cancer 2*. Fee WE, Goepfert H, Johns ME and Ward PH (eds), pp. 77–82. BC Decker: Toronto

De Vries EGE, Müller M, Meijer C, Jansen PLM and Mulder NH (1995) Role of the glutathione S-conjugate pump in cisplatin resistance. *J Natl Cancer Inst* **87**: 537-540

© Cancer Research Campaign 1998

Feller N, Broxterman HJ, Währer DCR and Pinedo HM (1995) ATP-dependent efflux of calcein by the multidrug resistance protein (MRP): no inhibition by intracellular glutathione depletion. *FEBS Lett* **368**: 385–388

Flens MJ, Izquierdo MA, Scheffer GL, Fritz JM, Meijer CJLM, Scheper RJ and Zaman GJR (1994) Immunochemical detection of multidrug resistance-associated protein MRP in human multidrug-resistant tumour cells by monoclonal antibodies. *Cancer Res* **54**: 4557–4563

Goto S, Yoshida K, Morikawa T, Urata Y, Suzuki K and Kondo T (1995) Augmentation of transport for cisplatin-glutathione adduct in cisplatin-resistant cancer cells. *Cancer Res* **55**: 4297–4301

Hayes JD and Wolf R (1990) Review article. Molecular mechanisms of drug resistance. *Biochem J* **272**: 281–295

Hermsen MAJA, Joenje H, Arwert F, Welters MJP, Braakhuis BJM, Bagnay M, Westerveld A and Slater R (1996) Centromeric breakage as a major cause of cytogenetic abnormalities in oral squamous cell carcinoma. *Genes Chromosom Cancer* **15**: 1–9

Hill BT (1996) Drug resistance: an overview of the current state of the art. *Int J Oncol* **9**: 197–203

Ishikawa T and Ali-Osman F (1993) Glutathione-associated cis-diamminedichloroplatinum(II) metabolism and ATP-dependent efflux from leukaemia cells. *J Biol Chem* **268**: 20116–20125

Ishikawa T, Bao JJ, Yamane Y, Akimaru K, Frindrich K, Wright CD and Ku MT (1996) Coordinated induction of MRP/GS-X pump and gamma-glutamylcysteine synthetase by heavy metals in human leukaemia cells. *J Biol Chem* **271**: 14981–14988

Ishikawa T, Wright CD and Ishizuka H (1994) GS-X pump is functionally overexpressed in cis-diamminedichloroplatinum(II)-resistant human leukaemia HL-60 cells and down-regulated by cell differentiation. *J Biol Chem* **269**: 29085–29093

Izquierdo MA, Shoemaker RH, Flens MJ, Scheffer GL, Wu L, Prather TR and Scheper RJ (1996*a*) Overlapping-phenotypes of multidrug resistance among drug unselected panels of human cancer cell lines. *Int J Cancer* **65**: 1–8

Izquierdo MA, Scheffer GL, Flens MJ, Giaccone G, Broxterman HJ, Meijer CJLM, Van Der Valk P and Scheper RJ (1996*b*) Broad distribution of the multidrug-resistance related vault protein LRP in normal human tissues and tumours. *Am J Pathol* **148**: 877–887

Izquierdo MA, Van Der Zee AGJ, Vermorken JB, Van Der Valk P, Beliën JAM, Giaccone G, Scheffer GL, Flens MJ, Pinedo HM, Kenemans P, Meijer CJLM, De Vries EGE and Scheper RJ (1995) Drug resistance-associated marker LRP for prediction of response to chemotherapy and prognosis in advanced ovarian carcinoma. *J Natl Cancer Inst* **87**: 1230–1237

Kelley DJ, Pavelic ZP, Gapany M, Stambrook P, Pavelic L, Gapany S, Gluckman JL (1993) Detection of P-glycoprotein in squamous cell carcinomas of the head and neck. *Arch Otolaryngol Head Neck Surg* **119**: 411–414

Kramer RA, Zakher J and Kim G (1988) Role of the glutathione redox cycle in acquired and de novo multidrug resistance. *Science* **24**: 694–697

Kurokawa H, Ishida T, Nishio K, Arioka H, Sata M, Fukumoto H, Miura M and Saijo N (1995) γ-Glutamylcysteine synthetase gene overexpression results in increased activity of the ATP-dependent glutathione S-conjugate export pump and cisplatin resistance. *Biochem Biophys Res Commun* **216**: 258–264

Loe DW, Deeley RG and Cole SPC (1996) Biology of the multidrug resistance-associated protein, MRP. *Eur J Cancer* **32A**: 945–957

Meijer C, Mulder NH, Timmer-Bosscha H, Sluiter WJ, Meersma GJ and De Vries EGE (1992) Relationship of cellular glutathione to the cytotoxicity and resistance of seven platinum compounds. *Cancer Res* **52**: 6885–6889

Mistry P, Kelland LR, Abel G, Sidhar S and Harrap KR (1991) The relationships between glutathione, glutathione S-transferase and cytotoxicity of platinum drugs and melphalan in eight human ovarian cell lines. *Br J Cancer* **6**: 215–220

Müller M, Meijer C, Zaman GJR, Borst P, Scheper RJ, Mulder NH, De Vries EGE and Jansen PLM (1994) Overexpression of the gene encoding the multidrug resistance-associated protein results in increased ATP-dependent glutathione S-conjugate transport. *Proc Natl Acad Sci USA* **91**: 13033–13037

Neuschwander-Tetri BA and Roll FJ (1989) Glutathione measurement by HPLC separation and fluorometric detection of glutathione-orthophtaldehyde adduct. *Anal Biochem* **176**: 236–241

Nooter K, Westerman AM, Flens MJ, Zaman GJR, Scheper RJ, Van Wingerden KE, Burger H, Oostrum R, Boersma T, Sonneveld P, Gratama JW, Kok T, Eggermont MM, Bosman FT and Stoter G (1995) Expression of the multidrug resistance-associated protein (MRP) gene in human cancers. *Clin Cancer Res* **1**: 1301–1310

O'Brien ML and Tew KD (1996) Glutathione and related enzymes in multidrug resistance. *Eur J Cancer* **32A**: 967–978

Scheffer GL, Wijngaard PLJ, Flens MJ, Izquierdo MA, Slovak ML, Pinedo HM, Meijer CJLM, Clevers HC and Scheper RJ (1995) The drug resistance-related protein LRP is the human major vault protein. *Nature Med* **1**: 578–582

Scheper RJ, Broxterman HJ, Scheffer GL, Kaaijk P, Dalton WS, Van Heijningen THM, Van Kalken CK, Slovak ML, De Vries EGE, Van Der Valk P, Meijer CJLM and Pinedo HM (1993) Overexpression of a M(r) 110,000 vesicular protein in non-P-glycoprotein-mediated multidrug resistance. *Cancer Res* **53**: 1475–1479

Taniguchi K, Wada M, Kohno K, Nakamura T, Kawabe T, Kawakami M, Kagotani K, Okumura K, Akiyama S and Kuwano M (1996) A human canalicular multispecific organic anion transporter (cMOAT) gene is overexpressed in cisplatin-resistant human cancer cell lines with decreased drug accumulation. *Cancer Res* **56**: 4124–4129

Vokes EE, Weichselbaum RR, Lippman SM and Hong WK (1993) Head and neck cancer. *N Engl J Med* **328**: 184–194

Welters MJP, Fichtinger-Schepman AMJ, Baan RA, Hermsen MAJA, Van Der Vijgh WJF, Cloos J and Braakhuis BJM (1997) Relationship between the parameters cellular differentiation, doubling time and platinum accumulation and cisplatin sensitivity in a panel of head and neck cancer cell lines. *Int J Cancer* **71**: 410–415

Yellin SA, Davidson BJ, Pinto JT, Sacks PG, Qiao C and Schantz SP (1994) Relationship of glutathione-S-transferase to cisplatin sensitivity in human head and neck squamous carcinoma cell lines. *Cancer Lett* **85**: 223–232

© Cancer Research Campaign 1998


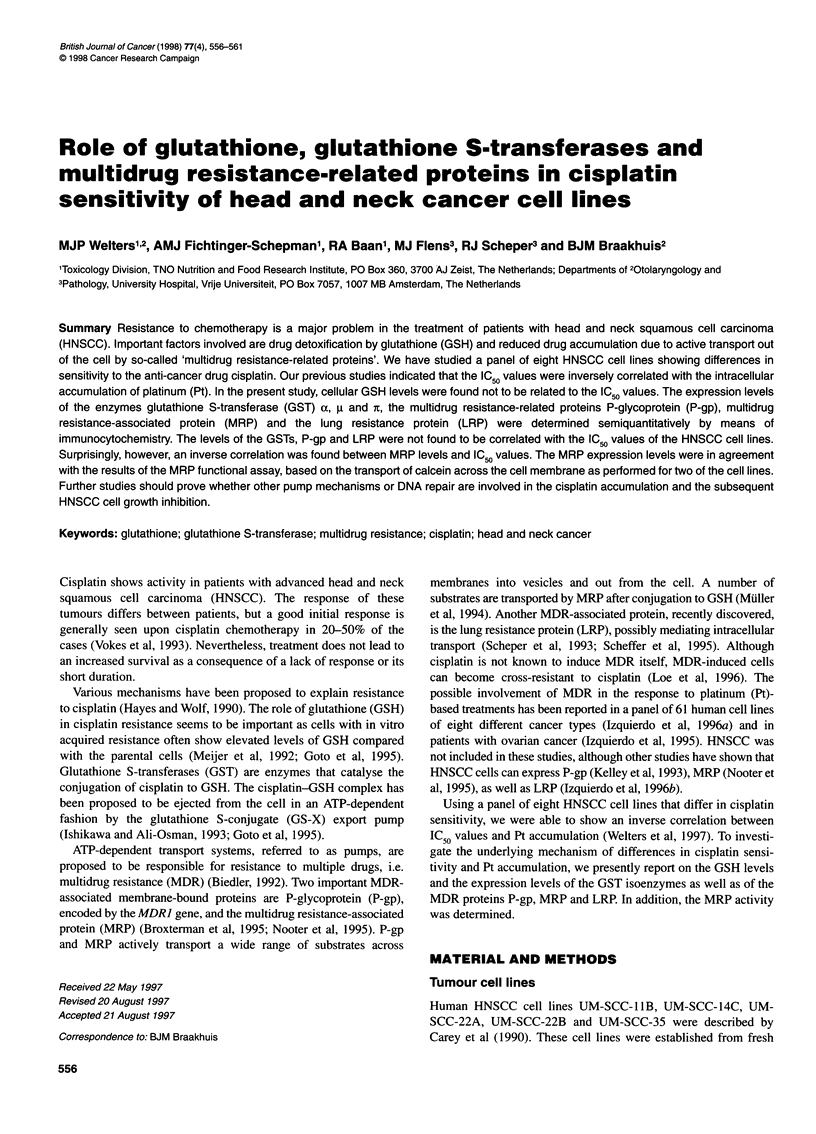

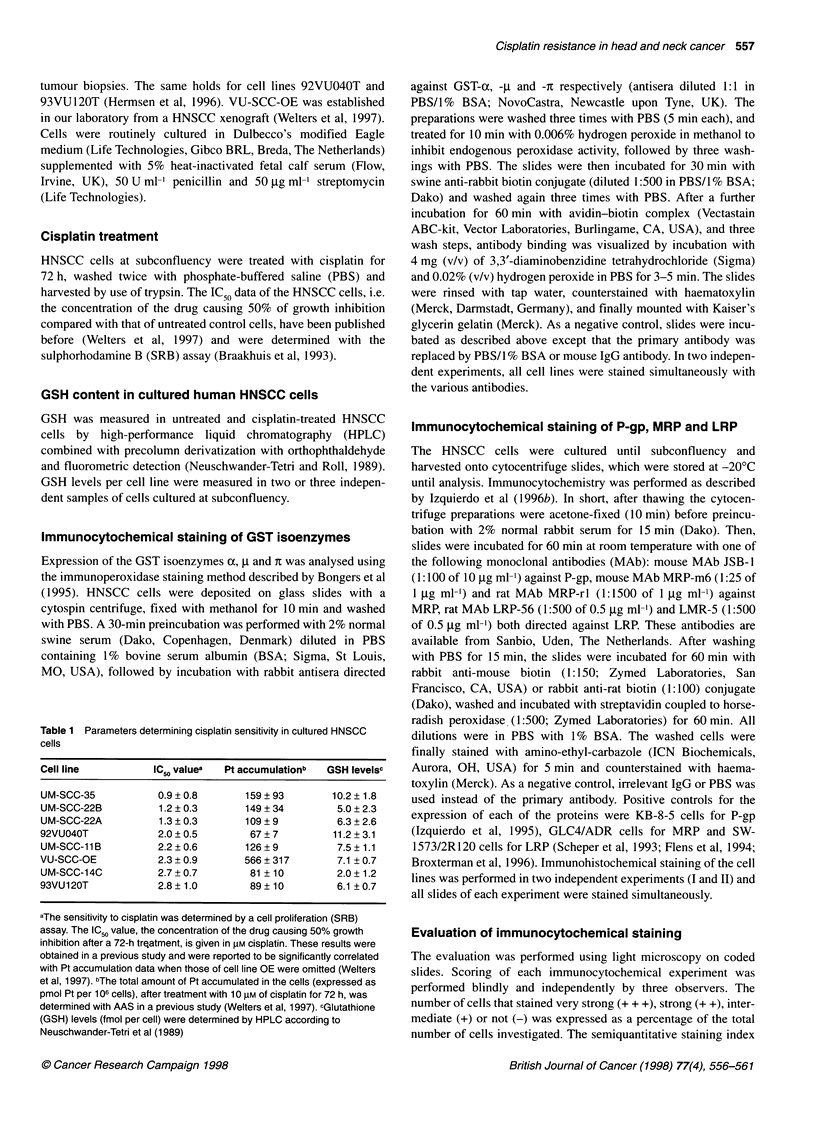

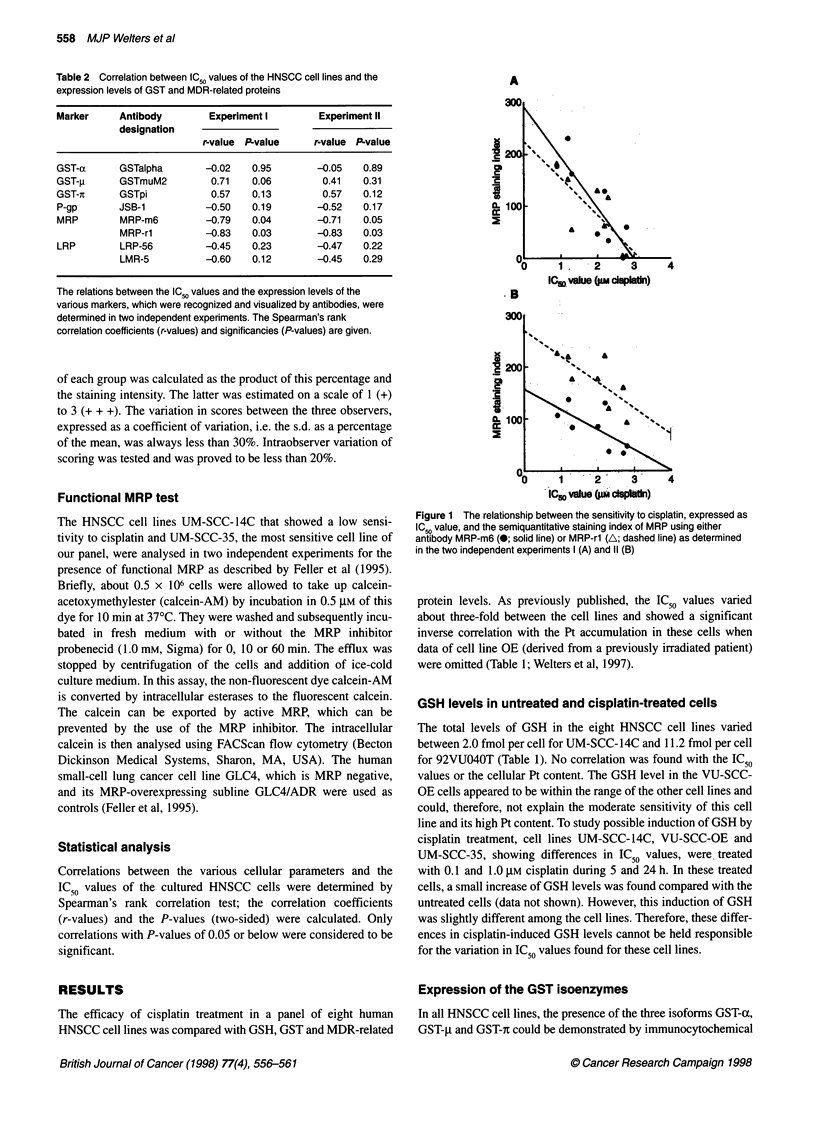

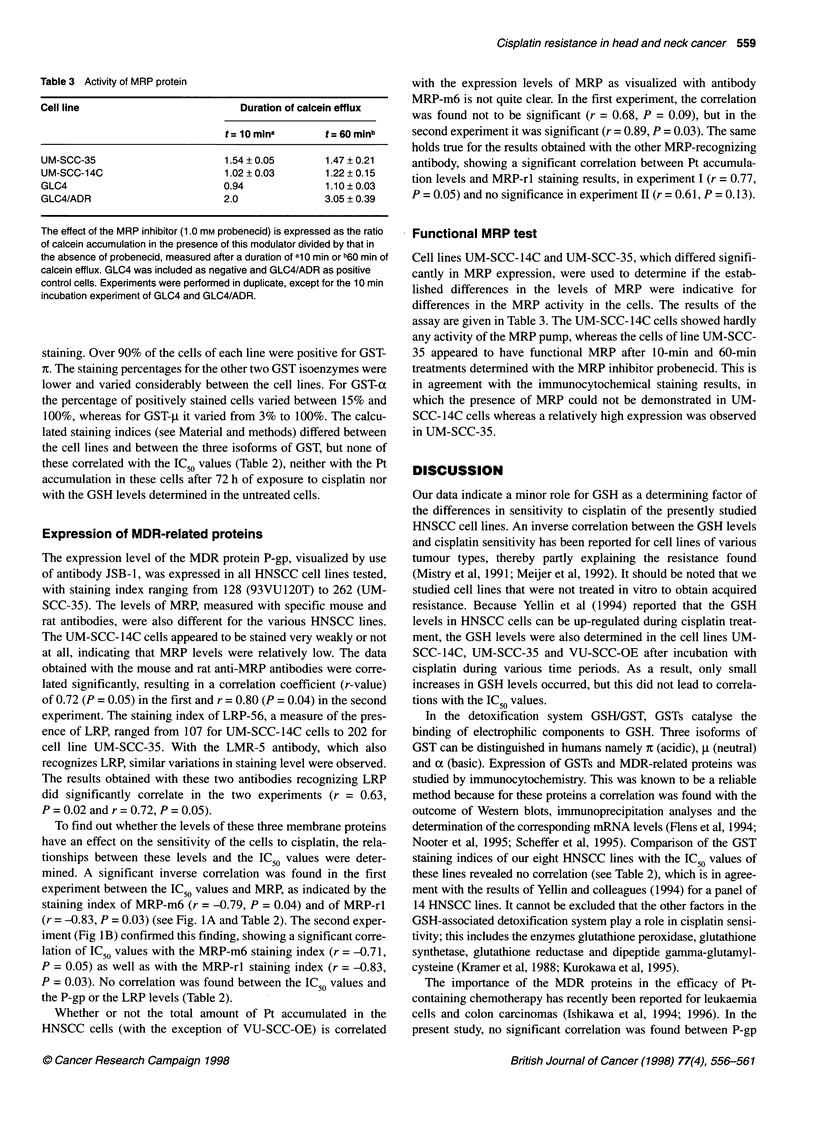

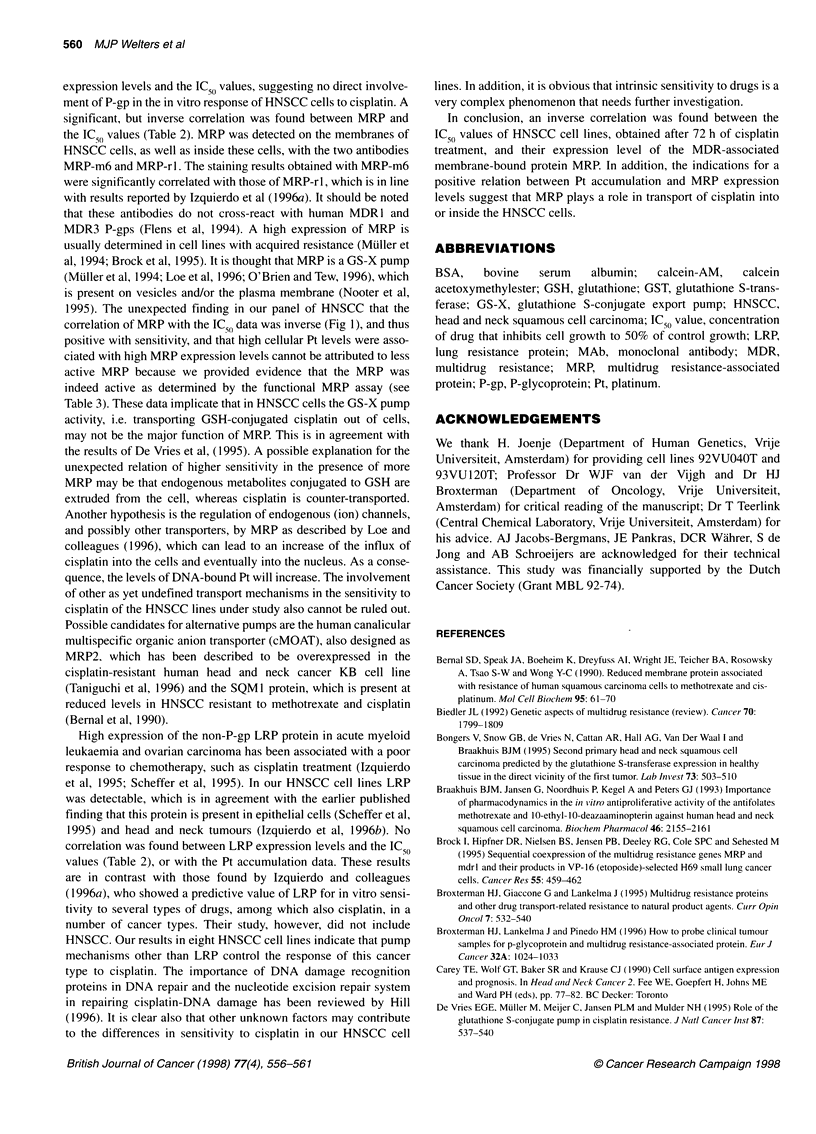

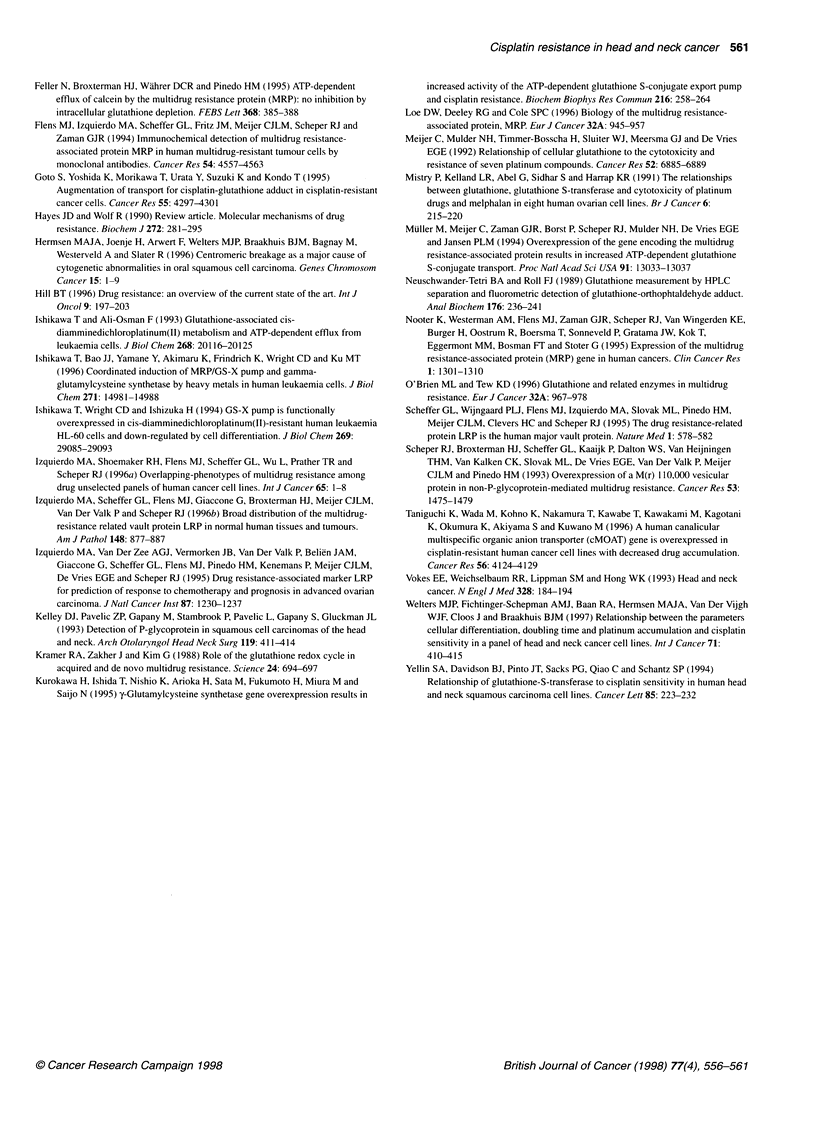


## References

[OCR_00613] Bernal S. D., Speak J. A., Boeheim K., Dreyfuss A. I., Wright J. E., Teicher B. A., Rosowsky A., Tsao S. W., Wong Y. C. (1990). Reduced membrane protein associated with resistance of human squamous carcinoma cells to methotrexate and cis-platinum.. Mol Cell Biochem.

[OCR_00620] Biedler J. L. (1992). Genetic aspects of multidrug resistance.. Cancer.

[OCR_00624] Bongers V., Snow G. B., de Vries N., Cattan A. R., Hall A. G., van der Waal I., Braakhuis B. J. (1995). Second primary head and neck squamous cell carcinoma predicted by the glutathione S-transferase expression in healthy tissue in the direct vicinity of the first tumor.. Lab Invest.

[OCR_00631] Braakhuis B. J., Jansen G., Noordhuis P., Kegel A., Peters G. J. (1993). Importance of pharmacodynamics in the in vitro antiproliferative activity of the antifolates methotrexate and 10-ethyl-10-deazaaminopterin against human head and neck squamous cell carcinoma.. Biochem Pharmacol.

[OCR_00637] Brock I., Hipfner D. R., Nielsen B. S., Jensen P. B., Deeley R. G., Cole S. P., Sehested M. (1995). Sequential coexpression of the multidrug resistance genes MRP and mdr1 and their products in VP-16 (etoposide)-selected H69 small cell lung cancer cells.. Cancer Res.

[OCR_00644] Broxterman H. J., Giaccone G., Lankelma J. (1995). Multidrug resistance proteins and other drug transport-related resistance to natural product agents.. Curr Opin Oncol.

[OCR_00649] Broxterman H. J., Lankelma J., Pinedo H. M. (1996). How to probe clinical tumour samples for P-glycoprotein and multidrug resistance-associated protein.. Eur J Cancer.

[OCR_00668] Feller N., Broxterman H. J., Währer D. C., Pinedo H. M. (1995). ATP-dependent efflux of calcein by the multidrug resistance protein (MRP): no inhibition by intracellular glutathione depletion.. FEBS Lett.

[OCR_00673] Flens M. J., Izquierdo M. A., Scheffer G. L., Fritz J. M., Meijer C. J., Scheper R. J., Zaman G. J. (1994). Immunochemical detection of the multidrug resistance-associated protein MRP in human multidrug-resistant tumor cells by monoclonal antibodies.. Cancer Res.

[OCR_00679] Goto S., Yoshida K., Morikawa T., Urata Y., Suzuki K., Kondo T. (1995). Augmentation of transport for cisplatin-glutathione adduct in cisplatin-resistant cancer cells.. Cancer Res.

[OCR_00684] Hayes J. D., Wolf C. R. (1990). Molecular mechanisms of drug resistance.. Biochem J.

[OCR_00688] Hermsen M. A., Joenje H., Arwert F., Welters M. J., Braakhuis B. J., Bagnay M., Westerveld A., Slater R. (1996). Centromeric breakage as a major cause of cytogenetic abnormalities in oral squamous cell carcinoma.. Genes Chromosomes Cancer.

[OCR_00699] Ishikawa T., Ali-Osman F. (1993). Glutathione-associated cis-diamminedichloroplatinum(II) metabolism and ATP-dependent efflux from leukemia cells. Molecular characterization of glutathione-platinum complex and its biological significance.. J Biol Chem.

[OCR_00704] Ishikawa T., Bao J. J., Yamane Y., Akimaru K., Frindrich K., Wright C. D., Kuo M. T. (1996). Coordinated induction of MRP/GS-X pump and gamma-glutamylcysteine synthetase by heavy metals in human leukemia cells.. J Biol Chem.

[OCR_00711] Ishikawa T., Wright C. D., Ishizuka H. (1994). GS-X pump is functionally overexpressed in cis-diamminedichloroplatinum (II)-resistant human leukemia HL-60 cells and down-regulated by cell differentiation.. J Biol Chem.

[OCR_00722] Izquierdo M. A., Scheffer G. L., Flens M. J., Giaccone G., Broxterman H. J., Meijer C. J., van der Valk P., Scheper R. J. (1996). Broad distribution of the multidrug resistance-related vault lung resistance protein in normal human tissues and tumors.. Am J Pathol.

[OCR_00728] Izquierdo M. A., van der Zee A. G., Vermorken J. B., van der Valk P., Beliën J. A., Giaccone G., Scheffer G. L., Flens M. J., Pinedo H. M., Kenemans P. (1995). Drug resistance-associated marker Lrp for prediction of response to chemotherapy and prognoses in advanced ovarian carcinoma.. J Natl Cancer Inst.

[OCR_00717] Karlsson M., Jungnelius U., Aamdal S., Boeryd B., Carstensen J., Kågedal B., Westberg R., Wingren S. (1996). Correlation of DNA ploidy and S-phase fraction with chemotherapeutic response and survival in a randomized study of disseminated malignant melanoma.. Int J Cancer.

[OCR_00735] Kelley D. J., Pavelic Z. P., Gapany M., Stambrook P., Pavelic L., Gapany S., Gluckman J. L. (1993). Detection of P-glycoprotein in squamous cell carcinomas of the head and neck.. Arch Otolaryngol Head Neck Surg.

[OCR_00740] Kramer R. A., Zakher J., Kim G. (1988). Role of the glutathione redox cycle in acquired and de novo multidrug resistance.. Science.

[OCR_00744] Kurokawa H., Ishida T., Nishio K., Arioka H., Sata M., Fukumoto H., Miura M., Saijo N. (1995). Gamma-glutamylcysteine synthetase gene overexpression results in increased activity of the ATP-dependent glutathione S-conjugate export pump and cisplatin resistance.. Biochem Biophys Res Commun.

[OCR_00751] Loe D. W., Deeley R. G., Cole S. P. (1996). Biology of the multidrug resistance-associated protein, MRP.. Eur J Cancer.

[OCR_00755] Meijer C., Mulder N. H., Timmer-Bosscha H., Sluiter W. J., Meersma G. J., de Vries E. G. (1992). Relationship of cellular glutathione to the cytotoxicity and resistance of seven platinum compounds.. Cancer Res.

[OCR_00760] Mistry P., Kelland L. R., Abel G., Sidhar S., Harrap K. R. (1991). The relationships between glutathione, glutathione-S-transferase and cytotoxicity of platinum drugs and melphalan in eight human ovarian carcinoma cell lines.. Br J Cancer.

[OCR_00766] Müller M., Meijer C., Zaman G. J., Borst P., Scheper R. J., Mulder N. H., de Vries E. G., Jansen P. L. (1994). Overexpression of the gene encoding the multidrug resistance-associated protein results in increased ATP-dependent glutathione S-conjugate transport.. Proc Natl Acad Sci U S A.

[OCR_00772] Neuschwander-Tetri B. A., Roll F. J. (1989). Glutathione measurement by high-performance liquid chromatography separation and fluorometric detection of the glutathione-orthophthalaldehyde adduct.. Anal Biochem.

[OCR_00777] Nooter K., Westerman A. M., Flens M. J., Zaman G. J., Scheper R. J., van Wingerden K. E., Burger H., Oostrum R., Boersma T., Sonneveld P. (1995). Expression of the multidrug resistance-associated protein (MRP) gene in human cancers.. Clin Cancer Res.

[OCR_00785] O'Brien M. L., Tew K. D. (1996). Glutathione and related enzymes in multidrug resistance.. Eur J Cancer.

[OCR_00789] Scheffer G. L., Wijngaard P. L., Flens M. J., Izquierdo M. A., Slovak M. L., Pinedo H. M., Meijer C. J., Clevers H. C., Scheper R. J. (1995). The drug resistance-related protein LRP is the human major vault protein.. Nat Med.

[OCR_00794] Scheper R. J., Broxterman H. J., Scheffer G. L., Kaaijk P., Dalton W. S., van Heijningen T. H., van Kalken C. K., Slovak M. L., de Vries E. G., van der Valk P. (1993). Overexpression of a M(r) 110,000 vesicular protein in non-P-glycoprotein-mediated multidrug resistance.. Cancer Res.

[OCR_00802] Taniguchi K., Wada M., Kohno K., Nakamura T., Kawabe T., Kawakami M., Kagotani K., Okumura K., Akiyama S., Kuwano M. (1996). A human canalicular multispecific organic anion transporter (cMOAT) gene is overexpressed in cisplatin-resistant human cancer cell lines with decreased drug accumulation.. Cancer Res.

[OCR_00811] Vokes E. E., Weichselbaum R. R., Lippman S. M., Hong W. K. (1993). Head and neck cancer.. N Engl J Med.

[OCR_00815] Welters M. J., Fichtinger-Schepman A. M., Baan R. A., Hermsen M. A., van der Vijgh W. J., Cloos J., Braakhuis B. J. (1997). Relationship between the parameters cellular differentiation, doubling time and platinum accumulation and cisplatin sensitivity in a panel of head and neck cancer cell lines.. Int J Cancer.

[OCR_00822] Yellin S. A., Davidson B. J., Pinto J. T., Sacks P. G., Qiao C., Schantz S. P. (1994). Relationship of glutathione and glutathione-S-transferase to cisplatin sensitivity in human head and neck squamous carcinoma cell lines.. Cancer Lett.

[OCR_00659] de Vries E. G., Müller M., Meijer C., Jansen P. L., Mulder N. H. (1995). Role of the glutathione S-conjugate pump in cisplatin resistance.. J Natl Cancer Inst.

